# Optimization of the method of ultrasonic extraction of lycopene with a green extract from the fruit of *Elaeagnus umbellata*, common in Western Georgia

**DOI:** 10.1002/fsn3.4030

**Published:** 2024-02-21

**Authors:** Nona Surmanidze, Maia Vanidze, Indira Djafaridze, Ruslan Davitadze, Inga Qarcivadze, Meri Khakhutaishvili, Aleko Kalandia

**Affiliations:** ^1^ Department of Chemistry, Faculty of Natural Sciences and Health Care Batumi Shota Rustaveli State University (BSU) Batumi Georgia

**Keywords:** *Elaeagnus umbellata*, green extraction, lycopene

## Abstract

The study determined the content of lycopene in the fruits of the *Elaeagnus umbellata* (35.25–60.21 mg/100 g), common at different heights above sea level in Western Georgia. For the effective extraction of lycopene as a biologically active substance, the optimal conditions for ultrasonic extraction were selected: sunflower oil was used as a “green solvent”; the ratio of solid mass and solvent was 1:50; temperature 30°C; ultrasound amplitude 40%; power 85 W; and extraction time 10 min. FTIR spectra revealed the characteristic functional groups of lycopene exhibiting two characteristic peaks at 2920 and 2950 cm^−1^. To explore the effect of lycopene on oil quality, the acid value, peroxide value, and p‐anisidine were determined in each oil sample. The antioxidant determination by inhibition of DPPH radicals showed significant differences in native oils and oils with lycopene.

## Graphical abstract

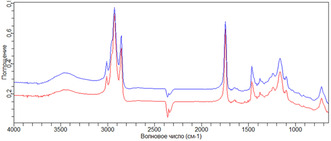

## 1 INTRODUCTION

The study of plant‐based substances rich in biologically active compounds has gained substantial traction in recent years, particularly due to their potential as preventive and therapeutic agents in a wide array of health conditions (Bacanli et al., 2017; Ganjhu et al., 2015; Ghias & Abdur, 2012; Guleishvili et al., 2018; Khattak, 2012; Sahakyan et al., 2019; Shonia et al., 2019; Who Library Cataloguing‐in‐Publication data, Regional Office for South‐East Asia, 2010; Zglińska et al., 2021, 2022). This relevance has been further underscored by the ongoing global health challenges such as the COVID‐19 pandemic (Galanakis, 2020; Galanakis et al., 2020; Ghellam et al., 2021). Plants' therapeutic potency is derived from their content of bioactive compounds, including alkaloids, carotenoids, glycosides, terpenoids, steroids, flavonoids, minerals, vitamins, organic acids, and others (Amorim et al., 2022; Fordham et al., 2001; Ghellam et al., 2021; Ghias & Abdur, 2012; Nazir et al., 2018; Surmanidze et al., 2021). Carotenoids, including β‐carotene, lycopene, lutein, phytoene, α‐cryptoxanthin, β‐cryptoxanthin, and others, are bioactive compounds commonly used for their antioxidant, immune‐boosting, and anticarcinogenic properties (Fordham et al., 2001; Khan et al., 2021; Nazir et al., 2018; Saini et al., 2022; Wu et al., 2011; Zglińska et al., 2021). Owing to their popularity, lycopene‐based pharmaceutical products have garnered considerable interest, spurring further research into the investigation and extraction of lycopene as a potent bioactive substance (Ghellam et al., 2021; Guo et al., 2009; Khan et al., 2021; Khattak, 2012; Wang, Hou, et al., 2020).

Lycopene, a naturally occurring carotenoid pigment, has attracted significant attention in recent years due to its extensive health benefits. Found primarily in red fruits and vegetables such as tomatoes, watermelons, carrots, grapefruits, papayas, and Elaeagnus (Bacanli et al., 2017; Ghias & Abdur, 2012), lycopene is an organic compound with the molecular formula C_40_H_56_ and a molecular weight of 536.85 g/mol (Amorim et al., 2022; Johnson, 2007). While insoluble in water, lycopene dissolves in certain organic solvents, making it suitable for various applications. This pigment plays a critical role in human biological systems, largely because of its remarkable antioxidant properties (Amorim et al., 2022; Patel, 2015; Przybylska, 2020; Wang, Chen, et al., 2020). By neutralizing free radicals and reducing oxidative stress, lycopene aids in the maintenance and promotion of overall health. Moreover, it has been found to be safe for consumption, with studies demonstrating its potential anticancer properties against gastric, breast, and prostate cancer cells, all without causing harm to normal human cells (Bacanli et al., 2017; Przybylska, 2020; Rahimi & Mikani, 2019).

The *Elaeagnus L*. family, and more specifically, the species *Elaeagnus umbellata*, has garnered considerable attention in the realm of scientific research. Globally, researchers are conducting in‐depth studies into these plants, driven by their potential in offering promising therapeutic and preventative health benefits (Gamba et al., 2020; Ishaq et al., 2015; Khanzadi, 2012; Kim et al., 2019; Nazir et al., 2018; Ozen et al., 2018; Patel, 2015; Spínola et al., 2019; Zubair et al., 2006).

Originating from Southern Europe and parts of Western and Central Asia, the autumn olive fruit (*Elaeagnus umbellata*) is a flavorful red berry that has recently found its way to various corners of the globe. Rich in numerous phytonutrients (Nazir et al., 2018; Spínola et al., 2019; Wang & Fordham, 2007; Zglińska et al., 2021), these berries are particularly notable for their lycopene content, which can reach 54 mg in 100‐g fresh fruits, 15 times higher than the lycopene content in tomatoes (Ghellam et al., 2021; Guo et al., 2009; Khattak, 2012; Wang, Hou, et al., 2020). The appealing sweet–tart taste of these berries makes them suitable for fresh consumption, as well as an excellent ingredient in a variety of culinary applications such as preserves, fruit rolls, juices, and other food items (Ghellam et al., 2021).

*Elaeagnus umbellata*, the most prevalent species within the *Elaeagnaceae* family, is widely distributed across Georgia. No other species from this genus have been reported in the region. This plant has successfully acclimated to the local climatic conditions, thriving in areas such as the coastal zone of Western Georgia and the mountainous regions (Beridze et al., 2020; Kohri et al., 2002). Despite its prevalence, there is a significant knowledge gap in the scientific literature concerning the plant's chemical composition and utilization, beyond its application in traditional medicine (Surmanidze et al., 2021, 2022; Vanidze et al., 2017).

The extraction technique and conditions significantly influence the yield and quality of bioactive substances obtained from plant materials. Various methodologies have been suggested for the production and extraction of carotenoids, including lycopene (Amorim et al., 2022; Chemat et al., 2017; Li et al., 2022; Linares & Rojas, 2022; Luengo et al., 2014). The water insolubility of lycopene, however, poses a challenge to its nutritional potency and industrial viability (Collins et al., 2006; Rahimi & Mikani, 2019; Zglińska et al., 2022). Traditional extraction methods, which often involve the use of extensive amounts of organic solvents, are characterized by lengthy extraction periods and relatively low yields of lycopene (Chemat‐Djenni et al., 2010; Rahimi & Mikani, 2019). Factors such as elevated temperatures (above 80°C), light exposure, oxygen, and extended exposure times can result in lycopene degradation (Rahimi & Mikani, 2019). Hence, it is crucial to select and implement an environmentally sustainable extraction method. Such “green” methods have been demonstrated to be more productive, cost‐effective, and environmentally friendly (Chemat‐Djenni et al., 2010). These methods typically encompass ultrasound‐assisted extraction (UAE), microwave‐assisted extraction (MAE), or high hydrostatic pressure‐assisted extraction (HHPAE) (Chemat et al., 2017).

Recent scientific studies highlight the extensive application of ultrasonic extraction as a cutting‐edge technique for lycopene extraction (Chemat et al., 2017; Li et al., 2022; Linares & Rojas, 2022). When ultrasound propagates through a liquid medium or solvent, it induces the formation of cavitation bubbles. These bubbles increase in number as they travel and eventually implode. This cavitation effect facilitates the rapid transfer of the maximum quantity of intracellular substances into the liquid (Luengo et al., 2014). Although hexane is a commonly used solvent for lycopene ultrasonic extraction due to its effectiveness, vegetable oil presents an intriguing alternative with notable benefits over hexane (Li et al., 2022; Rahimi & Mikani, 2019). Primarily, vegetable oil is an eco‐friendly solvent that yields a nondenatured and environmentally friendly product while requiring less energy for extraction. Additionally, it forms an oxygen barrier that helps prevent lycopene oxidation (Linares & Rojas, 2022; Rahimi & Mikani, 2019).

In Georgia, Elaeagnus is predominantly consumed in its unprocessed form. Nevertheless, it is infrequently processed as a lycopene‐containing raw material (Ozen et al., 2018; Zglińska et al., 2021). Consequently, our research aimed for the determination of the optimal conditions for obtaining a “green” extractant from *Elaeagnus umbellata* Thunb fruit, common in Western Georgia, by ultrasonic extraction of a lycopene‐containing preparation and detection of its qualitative characteristics; and determination of the antioxidant activity of the obtained extracts by the DPPH method.

## 2 MATERIALS AND METHODS

*Elaeagnus umbellata* fruits were collected from the coastal region of Western Georgia (200 m above sea level) in locations such as Kobuleti (coordinates to be provided: e.g., 41°48′40″ N 41°46′31″ E), Khelvachauri (41°58′25″ N, 41°65′83″ E), and Adjaristskali (41°54′44″ N, 41°72′96″ E), as well as from mountainous areas (200 m above sea level) in Keda (41°57′97″ N, 41°88′46″ E), Shuakhevi (41°62′96″ N, 42°19′12″ E), and Khulo (41°64′08″ N, 42°31′19″ E).

Ripe berries were picked manually during October and November (2015–2019), with 500 g collected for each replication. The fruits were randomly selected from five different trees, with each tree representing one biological replication, and were subsequently randomized.

After harvesting, the fruits underwent a selection process to eliminate any visible defects prior to analysis. The samples were lyophilized and crushed to a particle size not exceeding 250 μm. These samples were then stored in a refrigerator at 4°C until they were used.

The following solvents have been used for analysis: n‐hexane (Merck, 1.04391.2500, Germany, CAS‐No: 110‐5403), acetic acid (Merck, 1.00063.2511, Germany, CAS‐No: 64‐19‐17), methanol (Roth, Germany, Art.‐Nr. 8388.6), p‐anisidine (Germany, Aldrich, A88255‐100G, Lot # BCCB8823), 2, 2, 4 –tri methyl pentane (Acros organics, Code: 265440010, Lot: A0350543), and potassium hydroxide (Fisher chemical, Cod: P/5600/53, Lot: 1225333).

Ultrasound‐assisted extraction (UAE) was done in an ultrasonic probe processor (hielscher UP400St (400 W, 24 kHz)). The ultrasonic probe, equipped with a sonoprobe (18 mm), in pulsed mode affords an ultrasonic power while immersed into the solution for UAE (Rahimi & Mikani, 2019).

For determining the absorption spectra of the extracted lycopene content, Mettler Toledo UV/Vis spectrophotometer (UV 5) in cells with 1‐cm path length against the blank was exploited.

The nature of extracted lycopene was measured using the FTIR characterization method between 400 and 4000 cm^−1^ before and after UAE. All formulations of infrared spectra were organized employing Fourier transform infrared spectroscopy (Agilent Technologies, Cary 630 FTIR) (Rahimi & Mikani, 2019).

Samples were treated using an ultrasonic processor, and sunflower commercial oils were used as alternative solvent. The amplitude level range was 30%–40%, the power 85 W, the extraction time 10–20 min, 20 kHz of frequency, and pulsed operating mode (30s on/30s off). The temperature was constantly controlled with an external water bath to avoid temperatures over 30°C. After the extraction process, samples were centrifuged at 15,000 *g* for 10 min at 4°C and removed particulate residues before lycopene analysis. The lycopene‐enriched oils were collected.

Total lycopene amount in sunflower oil was expressed as absolute content mg lycopene/100 g of freeze‐dried tissue.

Qualitative analysis of lycopene was done by using ultra‐high‐performance (pressure) liquid chromatography (UHPLC), PDA, and MS method (Сaroten pos 15 min 03 ACN 500). Chromatographic column Acquity UPLC BEH C18, 1.7 m, solvent system: acetonitrile: methanol 7:3 (solvent A), and 100% water (solvent B) were used for the separation of compounds with the temperature range of 25–32°C and 5‐μL injection (Rivera et al., 2014).

**FIGURE 1:**
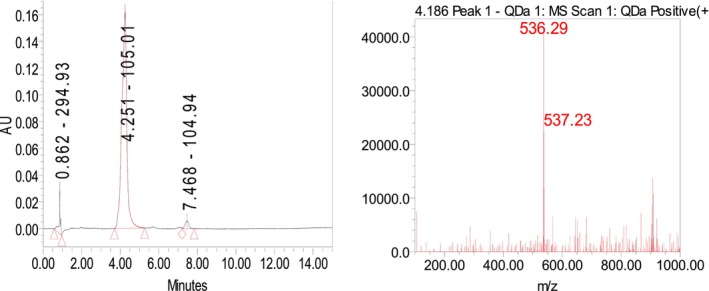
UHPLC‐PDA‐MS spectrum of lycopene.

**FIGURE 2:**
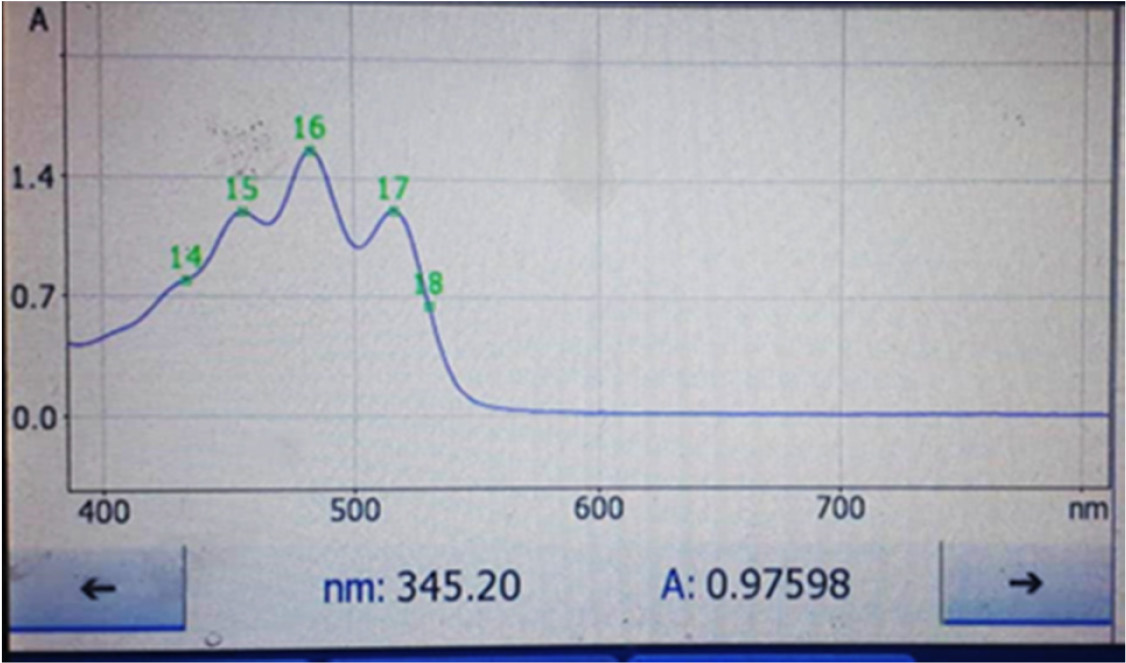
Ultraviolet absorption spectrum of lycopene extracted with hexane:acetone:methanol mixture (2:1:1) from the fruits of Elaeagnus taken in the coastal zone of the sea (0–200 m above sea level).

**FIGURE 3:**
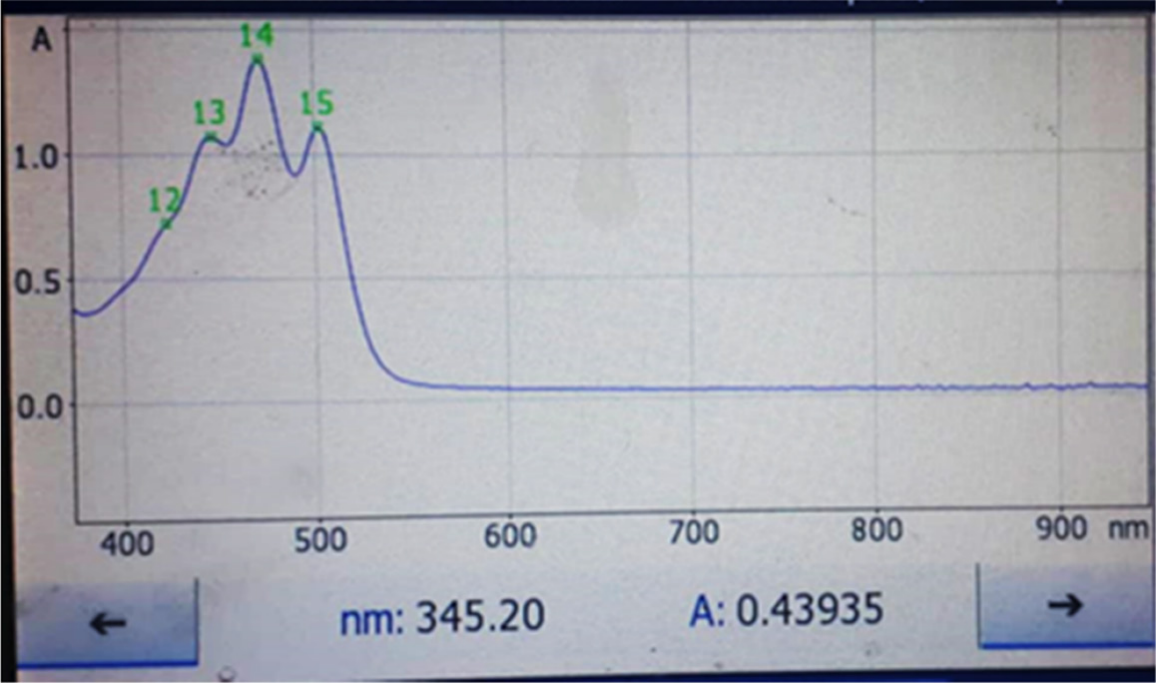
Ultraviolet absorption spectrum of lycopene extracted with hexane:acetone:methanol mixture (2:1:1) from Elaeagnus fruits taken in high mountain region (200 m above sea level).

**FIGURE 4:**
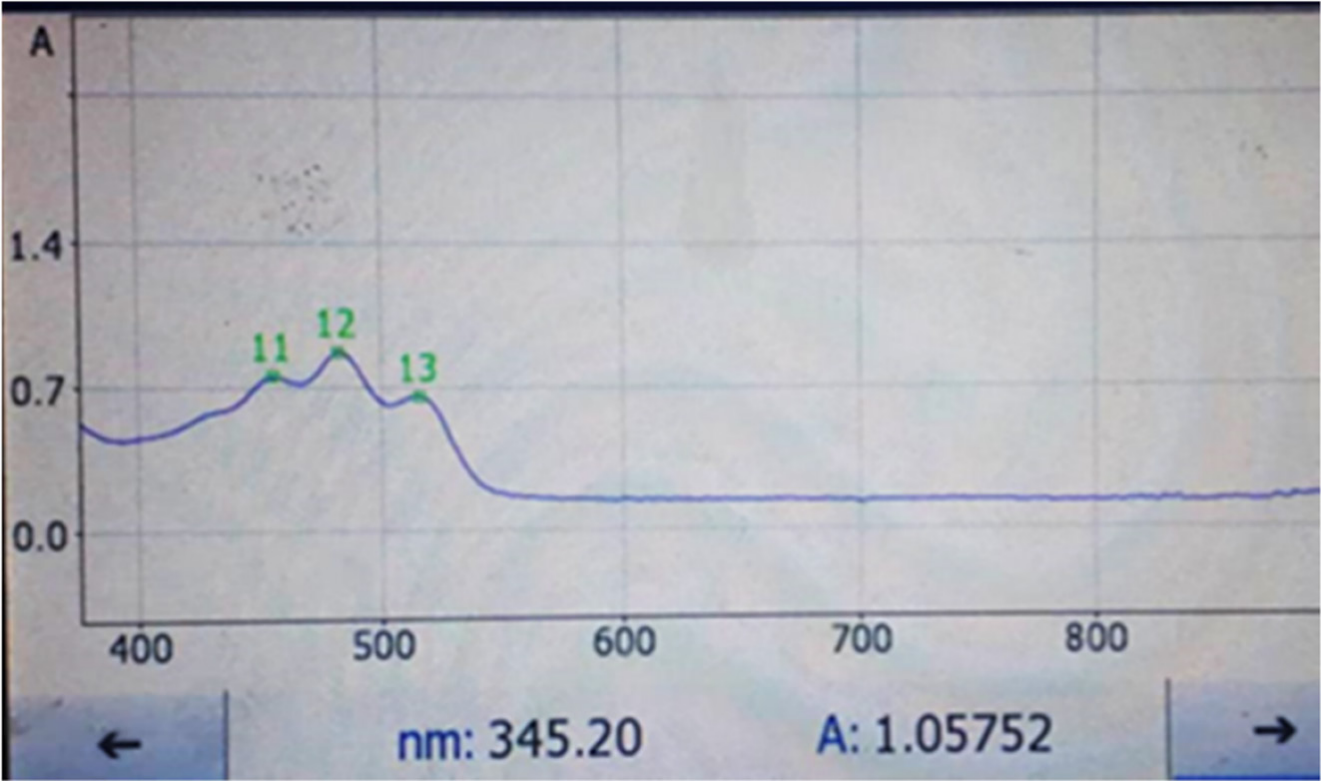
Ultraviolet absorption spectrum of lycopene extracted with sunflower oil from fruits of Elaeagnus collected in the coastal zone of the sea (0–200 m above sea level).

Total lycopene amount in the raw material (identified as 100%) was extracted using the mixture solvents hexane: methanol: acetone volume (2:1:1). Lycopene concentration of the extracting solvents was quantified by spectrophotometric measurement in the wavelength range of 350–600 nm (445, 472, and 502 nm) (Figures 1, 2, 3, 4).

To explore the effect of lycopene on oil quality, the acid value, peroxide value, and p‐anisidine were determined in each oil sample with international standard methods (ISO 660, ISO 3960, ISO 6885) (Wang, Chen, et al., 2020).

The antioxidant activity of oil samples was determined by the DPPH method. The results are given as 50% inhibition mg of sample, which is the amount of sample that inhibits the DPPH radical by 50% (the smaller the amount of sample is required for radical bleaching (mg), the higher the antioxidant activity is) (Gabour Sad et al., 2021; Kharadze, Djaparidze, et al., 2018; Kharadze, Japaridze, et al., 2018).

## 3 RESULTS

Using UHPLC‐PDA‐MS methods, lycopene was identified in the fruit of *Elaeagnus umbellata* [M]^+^ with a mass of *m/z* 536.48, a chromatogram retention time of 4.251, and an absorption maximum of 472.4 nm (Figure 1).

The quantitative content of lycopene was determined in the fruits of Elaeagnus, common at different altitudes above sea level, by the method of traditional extraction with a mixture of hexane:acetone:methanol (2:1:1).

The results of the study are shown in Table 1. The content of lycopene in lowland samples ranges from 35.25 to 37.58 mg/100 g, and in high mountain samples, it ranges from 46.08 to 60.21 mg/100 g.

**TABLE 1:** Yield of lycopene upon extraction mg/100 g with a mixture of hexane:acetone:methanol (2:1:1).

Height above sea level	Location name	Yield of lycopene mg/100 g under extraction conditions with a mixture of hexane:Acetone:Methanol
Up to 0–200 m	Kobuleti	35.25 ± 0.87
	Khelvachauri	33.15 ± 0.94
	Adjaristskali	37.58 ± 0.95
From 200 m above	Keda	46.08 ± 0.88
	Shuakhevi	49.58 ± 0.97
	Khulo	60.21 ± 0.98

Lycopene extraction was performed by ultrasound‐assisted extraction using sunflower oil as a solvent. First, a parametric study of ultrasound‐assisted extraction was done to determine the value of the optimum values of temperature and extraction time and the optimum solid/solvent ratio. Sunflower oil was used as an alternative solvent.

A sample of powdered *Elaeagnus umbellata* peels was mixed with 100‐mL solvent to produce different solid/solvent ratios (1:100, 2:100, and 3:100). During the extraction process, the sample was held in a thermostat‐controlled water bath. The extracts were collected at 5, 10, and 15 min.

The extraction temperature, ultrasonic power, and amplitude level were varied between 20 and 50°C, 60–120 W, and 20% and 60%, respectively. Lycopene contents of the samples were determined by spectrophotometric method. Extraction yield was defined as the percent ratio of the total weight of carotenoids extracted to the dry peels sample weight.

The maximum yield of lycopene, extracted from the fruit of Elaeagnus by the ultrasonic method, was at a ratio of solid mass to solvent of 2:100. Along with an increase in the mass of raw materials, the yield of lycopene decreased by 30% (34.53 mg/100 g), the extraction was effective in the pulsed mode, compared with the continuous mode, the optimal temperature for the extraction of lycopene was determined to be 30°C and ultrasonic power of 85 W.

**FIGURE 5:**
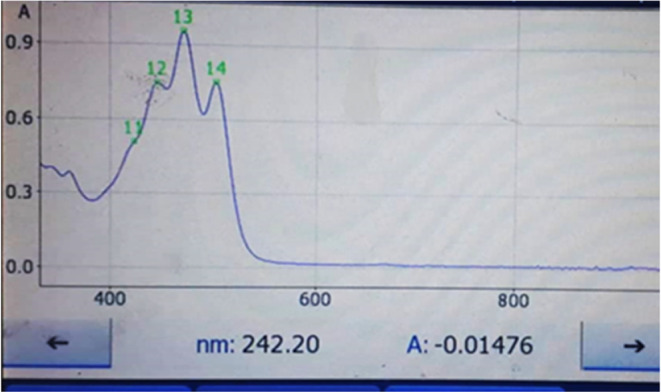
Ultraviolet absorption spectrum of lycopene extracted with sunflower oil from fruits of Elaeagnus collected in a high mountain region (200 m above sea level).

The content of extracted lycopene in vegetable oil was compared with the total content of lycopene in the feedstock (theoretical yield of extractable solvents hexane:methanol:acetone in volume (2:1:1)). For comparison, samples with a high content of lycopene were selected from both samples (sample 1 – fruits from Adjaristskali (up to 0–200 m) and sample 2 – fruits from Khulo (above 200 m)) (Figures 2, 3, 4, 5).

**FIGURE 6:**
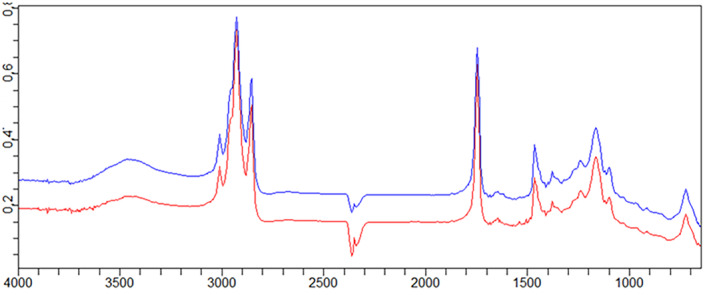
Infrared spectrum of lycopene extracted from Elaeagnus fruits with sunflower oil (sample 1 – Adjaristskali fruits (up to 0–200 m) and sample 2 – Khulo fruits (above 200 m)).

With sunflower oil, the yield of lycopene during extraction under optimal ultrasonic extraction conditions was about 85% of the total content, in one case 32.23 mg/100 g (85.76%), and in the other case 52.0 mg/100 g (86.36%) (Table 2).

**TABLE 2:** Percent yield of lycopene extracted from Elaeagnus fruits with sunflower oil.

Sample—Elaeagnus fruit	Yield of lycopene mg/100 g		Extraction of lycopene in sunflower oil %
	Under extraction conditions with a mixture of hexane:acetone:methanol	Under extraction conditions with sunflower oil	
Up to 0–200 m, sample from Adjaristskali	37.58	32.23	85.76
Above 200 m, sample from Khulo	60.21	52.0	86.36

To identify lycopene in lycopene‐enriched oil, we scanned the sample in infrared. The 1468 cm^−1^ peak was correlated to CH_2_ bending vibrations of lipids. Sunflower oil was used in the ultrasonic extraction of lycopene from *Elaeagnus umbellata* fruits, with the C–H stretching mode corresponding to the peaks 2920 cm^−1^ and 2950 cm^−1^. The structure of lycopene was related to these peaks (Figure 6).

To examine the effect of ultrasound on oil quality, the untreated sunflower oil and the oil treated with ultrasound at the optimum conditions were analyzed for acid value, peroxide value, p‐anisidine, and antioxidant activity. These characteristics were also determined during the oil storage period (1–3 months) (1, 2, 3, 4).

**DIAGRAM 1:**
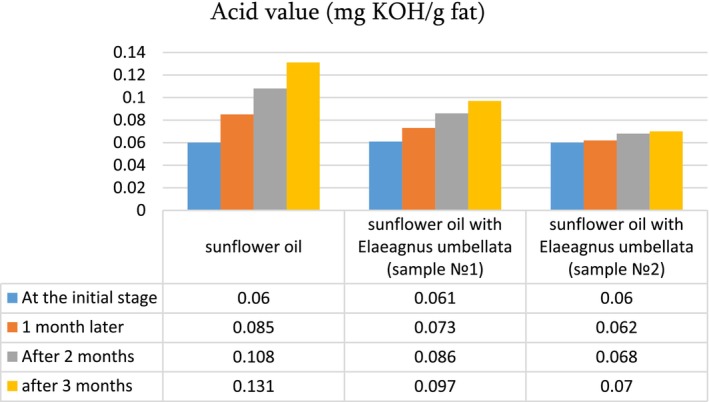
Acid value (mg KOH/g fat) of sunflower oil and sunflower oil enriched with lycopene during storage.

The acid number of the control oil sample and the oil sample enriched with lycopene is almost the same: 0.06–0.061 mg KOH/g fat. The peroxide index in the oil is 1.20 meq/kg, and in the lycopene‐enriched oil of Elaeagnus, it is 1.25 (sample 1) and 1.21 (sample 2) meq/kg.

A similar relationship is observed between the indicators of N‐anisidine: control sample – 3.5, oil of Elaeagnus enriched with lycopene – 3.7 (sample 1) and 3.6 (sample 2) (Table 3).

**TABLE 3:** Physical and chemical parameters of sunflower oil and sunflower oil enriched with lycopene.

Qualitative characteristics	Qualitative characteristics of sunflower oil and sunflower oil enriched with lycopene		
	Control sample of sunflower oil	Elaeagnus sunflower oil (sample 1), enriched with lycopene	Elaeagnus sunflower oil (sample 2), enriched with lycopene
Acid number (mg KOH/g fat)	0.060	0.061	0.060
Superoxide number meq/kg	1.20	1.25	1.21
N‐anisidine	3.5	3.7	3.6
Antioxidant activity, mg sample amount for 50% inhibition of the radical	0.85	0.42	0.38

When determining the antioxidant activity, oil samples enriched with lycopene were characterized by high activity, almost two times higher compared to the control oil sample. In particular, the smaller the mass of the sample needed for 50% radical inhibition is, the more active the product is: in the case of oil enriched with lycopene, 0.42 (sample 1) and 0.38 (sample 2) mg, the oil was sufficient for 50% inhibition, while 0.85 mg control oil provided 50% radical inhibition (Table 3).

**DIAGRAM 2:**
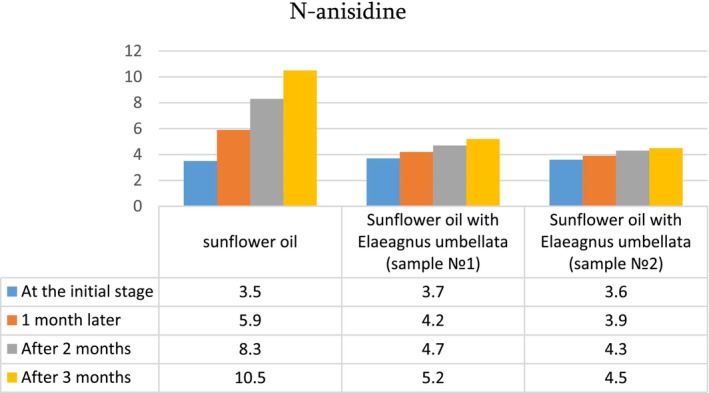
N‐anisidine value of sunflower oil and sunflower oil enriched with lycopene during storage.

In oil enriched with lycopene, quality indicators were determined within 3 months of storage with monthly control of characteristics. In sunflower oil (in the control sample), the acid number ranges from 0.06 to 0.131 units within 1–3 months, and in oil enriched with lycopene, the variability of acid number is less: 0.097 and 0.06–0.07 mg KOH/g fat (Diagram 1). A similar pattern of changes in indicators is observed in the case of N‐anisidine (Diagram 2).

**DIAGRAM 3:**
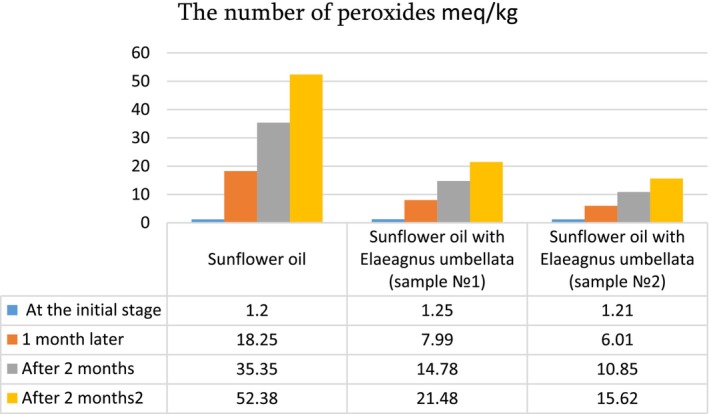
The number of peroxides of sunflower oil and sunflower oil enriched with lycopene during storage (meq/kg). The peroxide index significantly worsens (increases) during the storage period in the control oil sample (52.38 meq/kg); in oil enriched with lycopene, the result is positive, the indicator is almost two times less (15.82–21.48 meq/kg).

The peroxide index significantly worsens (increases) during the storage period in the control oil sample (52.38 meq/kg); in oil enriched with lycopene, the result is positive, the indicator is almost two times less (15.82–21.48 meq/kg) (Diagram 3).

**DIAGRAM 4:**
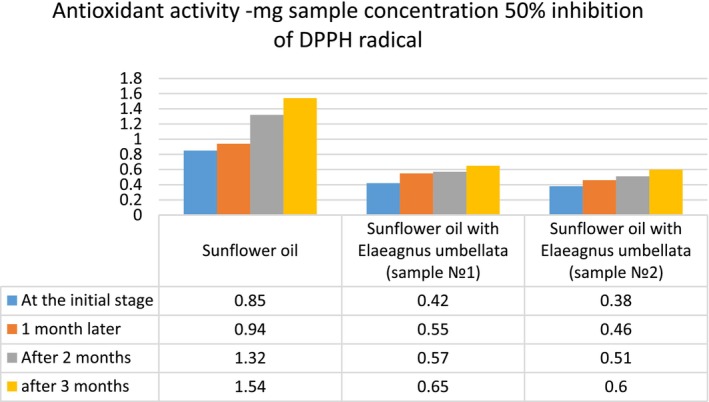
Antioxidant activity of sunflower oil and lycopene‐enriched sunflower oil during storage.

The antioxidant determination by inhibition of DPPH radicals showed significant differences in native oils and oils with lycopene. The level of antioxidant activity of oil enriched with lycopene is relatively stable within 3 months of storage. At the end of the storage period in the control sunflower oil, the activity decreases by almost two times (Diagram 4).

## 4 DISCUSSION

By selecting the optimal parameters for the extraction of lycopene with vegetable oil from the fruits of Elaeagnus by the method of ultrasonic extraction, it was possible to successfully extract lycopene. To extract lycopene from Elaeagnus fruits, ultrasonic extraction was chosen, and a green extractant, sunflower oil, was used as a solvent. The optimal conditions for ultrasonic extraction were determined as the ratio of solid mass and solvent 1:50, temperature 30°C, ultrasonic amplitude 40%, and power of 85 W. Extraction should be carried out in a pulsed mode – on (30 s) and off (30 s) with the extraction time of 10 min.

Compared to the theoretical yield, the yield of lycopene from Elaeagnus fruit in vegetable oil was approximately 85%. Infrared spectroscopy and spectroscopy in the ultraviolet and visible regions were used to identify and quantify lycopene in the resulting product. To compare the quality indicators of the product enriched with lycopene, at the initial and subsequent stages of storage, the following were determined: acid number (mg KOH/g fat), peroxide number meq/kg, n‐anisidine, and antioxidant activity.

Comparing the results obtained, we can conclude that the enrichment of sunflower oil with lycopene has a positive effect on the quality of the oil, namely, the curve of the change in acid number during the storage period is linear. It has also been confirmed that the oxidation processes in lycopene‐enriched sunflower oil are relatively slower than in the control sample.

The index of n‐anisidine was determined at the initial stage in all three samples by 3.5–3.7 units, after 3 months of storage in oil enriched with lycopene, the index of n‐anisidine was two times less (4.5–5.2) than in the control sample (10.5). The index of antioxidant activity of oil samples enriched with lycopene is almost two times higher than that of the control oil sample.

Based on the idea of “green chemistry”, the use of ultrasonic extraction and edible oil as a “green” extractant of lycopene from Elaeagnus fruit is an efficient and environmentally friendly approach.

It is planned for the future to obtain oil from *Elaeagnus umbellata* Thunb fruits and seeds using superfluid extraction, selection of optimal extractants and determination of their qualitative characteristics using infrared spectroscopy and gas–liquid chromatography (TRACE™ 1310 gas chromatograph—Thermo Scientific), and determination of the antioxidant activity of the obtained extracts and products using the DPPH method.

## AUTHOR CONTRIBUTIONS

**Nona Surmanidze:** Conceptualization (lead). **Maia Vanidze:** Supervision (lead). **Indira Djafaridze:** Methodology (lead). **Ruslan Davitadze:** Formal analysis (supporting). **Inga Qarcivadze:** Investigation (equal). **Meri Khakhutaishvili:** Investigation (equal). **Aleko Kalandia:** Supervision (lead).

## ACKNOWLEDGMENTS

Authors grateful to: Shota Rustaveli National Science Foundation of Georgia, Batumi Shota Rustaveli State University and Western Georgia Regional Chromatography Center for the support.

## FUNDING INFORMATION

The work has been carried out as part of a project with the financial support of Shota Rustaveli National Science Foundation (grant PHDF‐22‐2787).

## CONFLICT OF INTEREST STATEMENT

The authors declare no conflicts of interest.

## Data Availability

Research data are not shared.
